# Photodynamic Action of Curcumin and Methylene Blue against Bacteria and SARS-CoV-2—A Review

**DOI:** 10.3390/ph17010034

**Published:** 2023-12-25

**Authors:** Siu Kan Law, Albert Wing Nang Leung, Chuanshan Xu

**Affiliations:** 1 Guangzhou Municipal and Guangdong Provincial Key Laboratory of Molecular Target & Clinical Pharmacology, the NMPA and State Key Laboratory of Respiratory Disease, School of Pharmaceutical Sciences & Fifth Affiliated Hospital, Guangzhou Medical University, Guangzhou 511436, China; 2 Faculty of Science and Technology, The Technological and Higher Education Institute of Hong Kong, Tsing Yi, New Territories, Hong Kong; siukanlaw@thei.edu.hk; 3 School of Graduate Studies, Lingnan University, Tuen Mun, Hong Kong; albertleung@ln.edu.hk

**Keywords:** curcumin, methylene blue, photodynamic action, SARS-CoV-2

## Abstract

Coronavirus disease 19 (COVID-19) has occurred for more than four years, and the severe acute respiratory syndrome coronavirus 2 (SARS-CoV-2) causing COVID-19 is a strain of coronavirus, which presents high rates of morbidity around the world. Up to the present date, there are no therapeutics that can avert this form of illness, and photodynamic therapy (PDT) may be an alternative approach against SARS-CoV-2. Curcumin and methylene blue have been approved and used in clinical practices as a photosensitizer in PDT for a long time with their anti-viral properties and for disinfection through photo-inactivated SARS-CoV-2. Previously, curcumin and methylene blue with antibacterial properties have been used against Gram-positive bacteria, *Staphylococcus aureus* (*S. aureus*), and Gram-negative bacteria, *Escherichia coli* (*E. coli*), *Enterococcus faecalis* (*E. faecalis*), and *Pseudomonas aeruginosa* (*P. aeruginosa*). Methods: To conduct a literature review, nine electronic databases were researched, such as WanFang Data, PubMed, Science Direct, Scopus, Web of Science, Springer Link, SciFinder, and China National Knowledge Infrastructure (CNKI), without any regard to language constraints. In vitro and in vivo studies were included that evaluated the effect of PDT mediated via curcumin or methylene blue to combat bacteria and SARS-CoV-2. All eligible studies were analyzed and summarized in this review. Results: Curcumin and methylene blue inhibited the replication of SARS-CoV-2. The reactive oxygen species (ROS) are generated during the treatment of PDT with curcumin and methylene blue to prevent the attachment of SARS-CoV-2 on the ACE2 receptor and damage to the nucleic acids either DNA or RNA. It also modulates pro-inflammatory cytokines and attenuates the clotting effects of the host response. Conclusion: The photodynamic action of curcumin and methylene blue provides a possible approach against bacteria and SARS-CoV-2 infection because they act as non-toxic photosensitizers in PDT with an antibacterial effect, anti-viral properties, and disinfection functions.

## 1. Introduction

Coronavirus disease 19 (COVID-19) is caused by severe acute respiratory syndrome coronavirus 2 (SARS-CoV-2), which belongs to the subgenus sarbecovirus (previously lineage B) of genus betacoronavirus and occupies a unique phylogenetic position that is the most lethal [1]. This virus is capable of human-to-human transmission through droplets or direct contact, causing outbreaks of pneumonia and spreading around the world quickly [2,3]. There are no therapeutics that can avert this form of illness nowadays. Western medical approaches such as booster shots and variant-specific vaccines are used for the prevention and treatment of SARS-CoV-2 [4]. However, Western medicines such as remdesivir [5] and dexamethasone [6] may cause adverse drug reactions in some patients.

Meanwhile, photodynamic therapy has been developed for COVID-19 as an antimicrobial treatment that is safe and cost-effective. Antimicrobial photodynamic therapy (aPDT) is a process utilizing a light-activating photosensitizer (PS) that is usually applied in an oxygen-rich environment [7]. The energy of photons is absorbed by the photosensitizer and subsequently transferred to surrounding molecules for generating reactive oxygen species, causing damage to the macromolecules in microbes such as proteins, lipids, and nucleic acids, resulting in structural change and death [8].

Porphyrin is a common PS used to mitigate COVID-19 by preventing infections [9]. Protoporphyrin (PpIX) was also used as a sensor to monitor the presence of SARS-CoV-2 in the tissue, blood, urine, or feces can map the evolution and severity of the disease or monitor the response of COVID-19 to treatment modalities. This fluorescence spectroscopy has been applied as a diagnostic tool for COVID-19 with a low cost and high sensitivity. PDT with PpIX was effective in combating COVID-19 in the acute phase [10]. A series of water-soluble phosphorus(V) porphyrin molecules with OH or ethoxy axial ligands and phenyl/pyridyl peripheral substituents were used as antimicrobial agents in two Gram-negative bacteria models, such as *Escherichia coli* (*E. coli*) and *Acinetobacter baumannii* (*A. baumannii*). These porphyrins with an extremely low light dose of 5 J/cm^2^ in MIC_50_ values fight against COVID-19, based on the molecular interaction between phosphorus(V) porphyrin and bacterial lipid membranes [11]. The photodynamic therapy with Tetrahydroporphyrin-tetratosylate photodynamic therapy (THPTS) was also used to prevent the infection of COVID-19 with a virus load-reducing effect only at a higher concentration of 3 µM upon near-infrared light irradiation (760 nm, 3.9 or 7.8 J/cm^2^), which reduced the viral load in vivo, deactivated SARS-CoV-2 [12]. Thus, tetra-*p*-sulphonated-phenyl porphyrin (TSPP) was able to form cationic and J-aggregates at different pH values from 1 to 4, and concentrations around 10^−5^ M were suitable for the application of PDT to SARS-CoV-2 viruses [13].

Besides porphyrins, curcumin and methylene blue have also been widely used in the application of PDT against different types of microbes such as viruses, bacteria, protozoa, and fungi. Curcumin and methylene blue normally have limited adverse effect profiles and less damage to the host tissue with no resistance following multiple sessions of therapy [7]. This article aimed to review and discuss (i) the background of curcumin and methylene blue, (ii) the principles of PDT, (iii) photodynamic action on diverse organisms in (a) bacteria and (b) SARS-CoV-2 from COVID-19, and its clinical studies, as well as the current approach to nanotechnology in curcumin and methylene blue for the application of PDT.

## 2. Principles of PDT

PDT is a minimally invasive therapeutic modality used for the management of a variety of cancers and benign diseases. It is achieved with the use of visible or near-infrared irradiation to activate a light-absorbing compound (photosensitizer, PS) for producing singlet oxygen and other reactive oxygen species (ROS) that destroy the unwanted cells in the presence of molecular oxygen [14]. There are two types of photochemical action involved in the PDT as follows:

Type I: PS is excited in a triplet state reacting with biomolecules, which transfer the hydrogen atoms to produce the free radicals and radical ions for the generation of ROS in the presence of oxygen [15,16,17,18].

Type II: PS is excited in a triplet state reacting with oxygen in its triplet ground state to produce singlet oxygen (^1^O_2_) that is highly reactive and cytotoxic [15].

Type I and Type II reactions spontaneously occur at the same time, and the equilibrium between these two processes depends on the nature of the PS, the concentration of oxygen and substrate, and the affinity of the PS with the substrate.

Basically, photochemical action from curcumin and methylene blue as a PS consists of both Type I and Type II reactions. Curcumin has shown great potential as a PS in PDT because of its ability to absorb blue light (425–500 nm) for producing ROS to target cells or tissues [19]. Methylene blue can generate a high quantum yield of ^1^O_2_ via PDT to an infected area during excitation of the red light (630–680 nm) [20].

## 3. Curcumin

**Figure 1:**
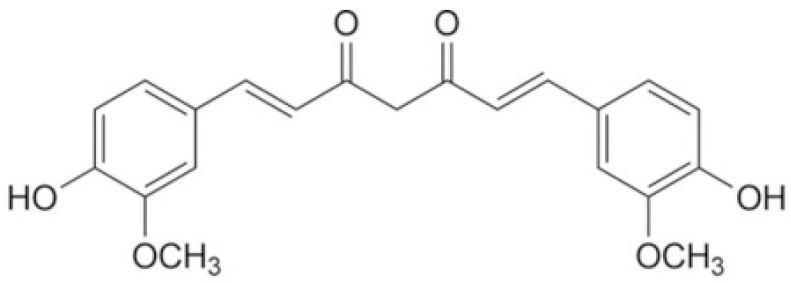
Chemical structure of curcumin.

Curcumin (Figure 1) is a natural substance extracted from *Curcuma longa* L. (turmeric) with the chemical structure of 1,7-bis(4-hydroxy-3-methoxyphenyl)-1,6-heptadiene-3,5-dione, (C_21_H_20_O_6_), consisting of phenol groups, ketone groups, and methoxy groups for a wide variety of biological activities [21]. It has been employed for the treatment of some diseases from the time of Ayurveda (1900 Bc) [22] because of numerous pharmacological effects including its antioxidant [23], anti-inflammatory [24], antiviral [25], antibacterial [26], antifungal [27], and anticancer properties [28]. Besides therapeutic activities, it is also used in the Indian subcontinent as a gold-colored spice, either in food preservation or as a yellow dye for textiles [22].

Curcumin has a broad absorption peak from 300 to 500 nm [29], and the maximum absorption peak can be divided into 408 to 430 nm in different solvent systems, such as tetrachloromethane and dimethyl sulfoxide. It is usually activated by light with a wavelength of around 425 nm to induce a strong phototoxicity at micromolar concentrations [30]. Currently, curcumin has extended its usage to antimicrobial activity for human clinical trials in a variety of conditions [31], acting as an antibacterial agent and exploiting about 100 different strains of both Gram-positive and Gram-negative pathogenic bacteria [32]. It has been used as a PS in the treatment of mouth pathogen microbes because of its photokilling and photo-biological abilities [29,33,34].

Curcumin is a suitable candidate as a PS in the application of PDT since it is a chemically pure substance with a specific uptake by the target tissue, minimal dark toxicity that is activated only upon irradiation, high photo-activity rate (quantum yield) to the production of ROS, and rapid clearance to avoid phototoxic side effects. It induces no toxic impact on healthy tissue and it should be able to be manipulated within short light intervals to ease treatment for outpatients [35].

## 4. Methylene Blue

**Figure 2:**
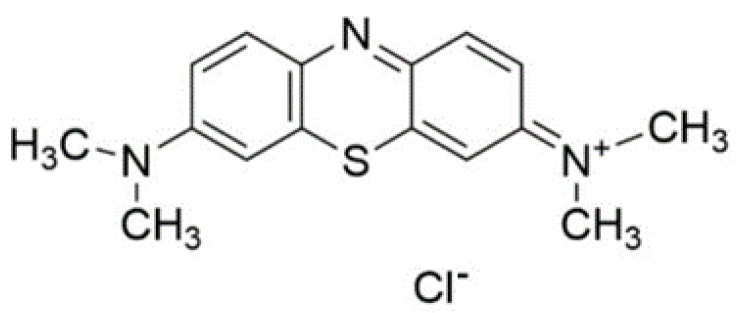
Chemical structure of methylene blue.

Methylene blue (Figure 2) is a heterocyclic aromatic compound belonging to the phenothiazine type with the chemical structure of 7-(Dimethyl-amino)phenothiazin-3-ylidene]-dimethyla-zanium chloride (C_16_H_18_ClN_3_S). It consists of two benzene rings attached to one nitrogen atom and one sulfur atom and was first synthesized as a textile dyestuff by Caro in 1876 [36]. Methylene blue has been used to stain the nervous tissue [37] as an analgesic [38] and antimalarial component [39]. Nowadays, methylene blue is a well-known dye in medicine and has been applied for topical treatment during photodynamic therapy (PDT) [40].

Since methylene blue has very low tissue toxicity, it has been used in medical practice for more than a hundred years, including the treatment of ifosfamide encephalopathy, methemoglobinemia, urolithiasis, and cyanide poisoning [41]. It is non-toxic and can be administered in high doses to humans orally or intravenously [42]. Methylene blue is possible to achieve optimal light penetration into tissue because of the strong light absorption at 620 nm wavelengths; thus, this is an excellent choice for superficial treatments of the skin or oral cavity [35]. Thus, methylene blue is safe and effective for use in PDT because of its ability to generate singlet oxygen with high light absorption at 665 nm against several diseases [43].

## 5. Comparing the Photodynamic Action of Methylene Blue and Curcumin

Methylene blue is an FDA-approved PS [44]; curcumin is not approved as a PS and is only available in the United States as a dietary supplement [45]. However, curcumin has been used as a photosensitizer in PDT for a long time, especially in clinical studies.

Methylene blue is a hydrophilic compound with a low molecular weight and a positive charge, which easily passes through Gram-positive and Gram-negative bacterial membranes. When being irradiated by a light source of approximately 630 nm, this compound promotes irreversible oxidation of the target cells [46]. Additionally, curcumin is a hydrophobic compound. It has a high molecular weight and a neutral charge and can be irradiated by a light source of approximately 425 nm [34,47]. Compared to methylene blue, it is insoluble in water with a shorter wavelength in the application of PDT. However, curcumin is a natural product without side effects. *Methylene blue is a cationic dye* that may cause some health problems such as asthma and eye, or skin irritations [48,49].

## 6. Photodynamic Action against Bacteria

Generally, an applied therapeutic *photodynamic* method involves the activation of curcumin and methylene blue with blue or red lights at specific wavelengths (425–500 nm and 630–680 nm, respectively).

Growing evidence has shown that curcumin possesses antibacterial properties in aPDT treatment against Gram-positive bacteria *Staphylococcus aureus* (*S. aureus*), and Gram-negative bacteria *Escherichia coli* (*E. coli*), *Enterococcus faecalis* (*E. faecalis*), and *Pseudomonas aeruginosa* (*P. aeruginosa*). The possible mechanism of aPDT for curcumin involves producing different types of ROS, such as hydrogen peroxide, hydroxide radical, superoxide, and singlet oxygen [50]. Cytotoxic ROS react with DNA, proteins, lipids, and other components to induce cell death in organisms [51]. The singlet oxygen produced has a very small radius of action, which is less than 0.02 μm, and damage only occurs in the presence of the photosensitizer and under photoactivation. Cell death of an organism is caused by the cell wall or membrane lysis and/or the inactivation of proteins or enzymes essential for microbial metabolism [52]. The photodynamic action of methylene blue against bacteria is similar to that of curcumin. Actually, curcumin and methylene blue are very safe, and their photodynamic actions have a broad spectrum of antibacterial effects and no bacterial resistance [53,54], the PDT with curcumin and methylene blue has been used in antibacterial treatment (Table 1 and Table 2).

## 7. Photodynamic Action against SARS-CoV-2

SARS-CoV-2 is a single-stranded RNA-enveloped virus that is 29,881 bp in length and encodes 9860 amino acids [80]. It breaks the plasma membrane, enters human cells, and exposes its genetic material, causing severe damage to human RNA and DNA [81]. The photodynamic action of porphyrin generates singlet oxygen (^1^O_2_), which has a diffusion distance of approximately 0.01 to 0.02 μm before quenching. Since PSs need to be closely associated with the target substrate for maximum impact, the affinity of a virus for the heme structure indicates high viral destruction. These titers are restricted to the zone of photoactivation [82].

Curcumin and methylene blue are other common PSs in the photodynamic treatment of SARS-CoV-2 (Table 3 and Table 4) that attempt to inactivate the virus. They have the ability to enter the host cell and bind to the DNA, RNA, as well as protein of the virus. When binding to DNA, RNA, or protein of the virus, cross-linking mechanics could be altered to cause damage to [83] the glycoprotein, resulting in the inhibition of viral replication [81]. Since SARS-CoV-2 requires an enveloped homotrimeric spike glycoprotein to interact with the cellular receptor ACE-2 [84] and the selective impairment of surface proteins, like spike glycoprotein, PDT can effectively inhibit the infectivity of SARS-CoV-2 [85].

## 8. Mechanism of Photodynamic Action against SARS-CoV-2

Methylene blue has photodynamic effects used in different illnesses, including cirrhosis of the liver, hypoxemia, and severe hepatopulmonary syndrome [92]. It reduces the oxidized ferric form of hemoglobin (Fe^3+^), which permanently binds oxygen to the ferrous (Fe^2+^) form. This improves hemoglobin’s ability to bind oxygen and, as a result, oxygen transport to tissues, which is beneficial for COVID-19 patients [93]. Silent hypoxemia is the term for the low oxygen levels that COVID-19 patients frequently exhibit. These levels are usually incompatible with a life without dyspnea [94].

Curcumin can improve COVID-19 patients’ prognoses by drastically reducing the length of common symptoms, hospital stays, and mortality [95]. It inhibits the ACE2 receptor and the SARS-CoV-2 S protein, as well as SARS-CoV-2 proteases such as papain-like protease and main protease [96,97]. Additionally, curcumin can dramatically lower inflammatory cytokine levels and stimulate anti-inflammatory pathways, which raises anti-inflammatory cytokine levels and partially restores the pro- and anti-inflammatory chemical balance in COVID-19 patients. This may help to control the cytokine storm associated with COVID-19 [96].

## 9. Clinical Study for COVID-19

Curcumin is widely found in food and it is a safe drug with broad prospects for clinical application [98]. The use of nanocurcumin is a successful practice, and effectively increases O_2_ saturation and reduces the severity of symptoms in COVID-19 patients [99]. The minor side effects of an intake of curcumin formulations include colds, irritation, indigestibility, and nausea, which in a few cases might be attributed to adjuvants and emulsifiers [100]. However, most of these studies are single-center studies with small populations and different oral doses of nanocurcumin. It is better to have more long-term, multi-center, and large-sample studies [96].

Methylene blue can also be used for COVID-19 treatment [101]. However, administration of methylene blue with meticulous consideration of the dosage is necessary to prevent any untoward effects, since methylene blue is a monoamine oxidase (MAO) inhibitor and can interact with antidepressants to cause severe toxicity of serotonin [102]. It has also been found to interact with dapsone to form hydroxylamine, which oxidizes hemoglobin and may cause hemolysis [103]. In patients with G6PD deficiency, this can be detrimental and may cause severe hemolysis [104]. Thus, this needs to be studied further to find the optimal dosage for clinical study.

## 10. Discussion

PDT, with high cure rates, minimal toxicity in healthy tissues, specific targeting and selectivity, and a low amount of side effects, shows potential for use as an adjuvant therapy when combined well with all other tumor interventions such as chemotherapy, surgery, radiotherapy, immunotherapy, or treatment of cancer [105]. However, curcumin as a PS used in the clinical application of PDT has some limitations, such as poor solubility, stability, and photostability in aqueous solutions, as well as rapid metabolism and systemic elimination [106]. Methylene blue is more soluble in water but its clinical use has been limited by the rapid enzymatic reduction in the biological environment [107].

These problems of curcumin and methylene blue could be overcome with the help of nanotechnology, which produces nanoscale curcumin or methylene blue; its physical specifications, chemical properties, and biological characteristics enable it to have great bioavailability, solubility, transport, and effectiveness [108]. Nanotechnology improves the characteristics of curcumin and endows it with the ability to target an infected area, as well as inhibit viral replications that prevent the TMPRSS2 from interacting with disintegrin metallopeptidase domain 17 (ADAM17) to give a high level of ACE2 expression, causing lung inflammation and pulmonary oedema [109].

The nanocurcumin could inhibit natural killer cells and T helper 17 cells, as well as reduce the release of inflammatory factors such as IL-1β, IL-6, and TNF-α. Since nanocurcumin may effectively increase O_2_ saturation and reduce the severity of symptoms in COVID-19 patients, the formulations might be used as a supplement to accelerate the recovery of patients, improve the symptoms, and shorten the recovery period of COVID-19 patients [99]. 

Additionally, methylene blue-embedded nano or microparticles could effectively deliver photo-responsive molecules to tissues and cells for translocating them into cellular compartments, thereby producing significant amounts of ROS in the target tissues. Methylene blue would maximize the therapeutic efficacy of PDT while reducing immunogenicity and side effects [110].

The solubility of curcumin and methylene blue could also be improved via the formation of nanogels, ranging from 10 to 100 nm [111]. These nanogels are composed of hydrogel and synthesized through either the physical or chemical cross-linking of polymers. The cross-linked structure provides a sustainable storage and release rate [112]. They are developed as carriers for curcumin and methylene blue delivery because the nanogels could absorb biologically active molecules spontaneously via the formation of salt bonds, hydrogen bonds, or hydrophobic interactions to enhance their bioavailability, such as by enhancing their solubility and stability.

Thus, PDT with nano-scale curcumin or methylene blue as a potential therapy against SARS-CoV-2 is still being investigated; but much more research work needs to be carried out, especially concerning safety assessments for human clinical trials.

## 11. Conclusions

The above information demonstrates that curcumin and methylene blue are two promising photosensitizers for PDT with an antibacterial effect, anti-viral properties, and disinfection function. It presents a possible against bacteria and SARS-CoV-2 infection. The photodynamic action of curcumin and methylene blue can be enhanced with the help of nanotechnology in future developments.

## Figures and Tables

**Table 1:** The photodynamic action of curcumin against bacteria.

	Study	Usage of Light and Energy (J)	Experiment Parameters	Conclusion	References
1	Antimicrobial photodynamic therapy with curcumin on methicillin-resistant *Staphylococcus aureus* biofilm	The strains are induced to form biofilm and incubated with curcumin for 20 min, irradiated with a Light-Emitting Diode at 450 nm with 50 J/cm^2^ for 455 s.	Colony-forming units, scanning electron microscopy, confocal microscopy images, and resazurin dye test.	PDT reduces the growth of the MRSA strain biofilm, making it a relevant alternative possibility for inactivation.	[55]
2	Antimicrobial photodynamic therapy (aPDT) with curcumin controls intradermal infection by *Staphylococcus aureus* in mice with type 1 diabetes mellitus: a pilot study	Curcumin is photoactivated ex vivo with LED light at 450 nm delivering a fluency of 13.5 J/cm^3^.	MRSA ATCC 43,300 strain infection study, immuno-assay in diabetes mellitus.	The therapeutic potential of PDT using curcumin when administered intradermally in the treatment of infections caused by *S. aureus* in mice with type 1 diabetes mellitus.	[56]
3	Photodynamic action of LED-activated curcumin against *Staphylococcus aureus* involving an intracellular ROS increase and membrane damage	*S. aureus* is incubated with different concentrations of curcumin for 60 min, then irradiated with blue light at 470 nm with a light dose of 3 J/cm^2^.	Colony-forming units, confocal microscopy images, flow cytometry, FCM analysis with DCFH-DA staining, transmission electron microscopy, and ROS assay.	Blue light-activated curcumin markedly damaged membrane permeability, resulting in cell death of *S. aureus*, and highlighted that an intracellular ROS increase might be an important event in the photodynamic killing of *Staphylococcus aureus*.	[57]
4	Bacterial viability after antimicrobial photodynamic therapy with curcumin on multi-resistant *Staphylococcus aureus*	Methicillin-resistant *S. aureus* strains and American-type culture collection (ATCC) of *Staphylococcus aureus* are evaluated in vitro, after incubation with curcumin for 20 min, and irradiated with LED light at 450 nm.	Colony-forming units, confocal microscopy images, and flow cytometry.	PDT with curcumin may be an interesting therapeutic alternative, because of its in vitro response, for the control of multi-resistant clinical *S. aureus* strains.	[58]
5	Antimicrobial photodynamic therapy using blue LED light for the activation of curcumin extracts (curcuma longa) on *Staphylococcus aureus* bacteria: an in vitro study	Blue LED light of 400–450 nm with an energy density of 16.19 J/cm^2^ is used to activate exogenous curcumin extracts in *S. aureus* in vitro.	Colony-forming units, confocal microscopy images, and flow cytometry.	LED irradiation of PDT can activate curcumin extracts to increase the percentage of *S. aureus* bacterial death (91.49 ± 0.01%) with curcumin extract and 44.88 ± 0.18% without curcumin extract.	[59]
6	Photodynamic therapy control of *Staphylococcus aureus* intradermal infection in mice	Blue LED light is associated with curcumin to treat *S. aureus* intradermal infection in mice at 450 nm with 75 mW/cm^2^, and 54 J/cm^2^ for 10 min.	Colony-forming units, confocal microscopy images, and flow cytometry.	PDT may be effective against MRSA infection in a murine model of intradermal infection.	[60]
7	Photokilling of bacteria via curcumin in different aqueous preparations: Studies on curcumin and curcuminoids XXXVII	The bacteria are exposed to 1 to 25 micron curcumin solubilized in DMSO, cyclodextrins, liposomes, and surfactants for 30 min, then are incubation irradiated with fluorescent tubes emitting blue light at 430 nm with 17 mW/cm^2^ and a radiant exposure of 0.5–30 J/cm^2^.	Colony forming units, fluorescence microscopy, and confocal microscopy images.	Aqueous preparations of DMSO, polyethylene glycol, and the copolymer poly(ethylene glycol)-block-poly(propylene glycol)-block-poly(ethylene glycol) are the most efficient vehicles for curcumin to exert photo killing of Gram-positive and Gram-negative bacteria.	[61]
8	Potentiation of antimicrobial photodynamic therapy via curcumin-loaded graphene quantum dots	Blue LED light is associated with 100 µm of curcumin at 405 nm in the irradiance of 30 J cm^−2^ to NIH/3t3 fibroblasts.	Colony-forming units, cytotoxicity assay, and ROS assay.	Curcumin-loaded graphene quantum dots enhance antimicrobial photodynamic effects and can be used as an alternative effective treatment for resistant infections.	[62]
9	Formulation and characterization of lyophilized curcumin solid dispersions for antimicrobial photodynamic therapy (aPDT): Studies on curcumin and curcuminoids LII	Blue LED light is associated with 0.5 to 10 µM low concentrations of curcumin at 450 nm in the irradiance of 11–16 J/cm^2^ to *E. faecalis* and *E. coli*.	Colony-forming units, cytotoxicity assay, differential scanning calorimetry, photostability, and thermal stability tests.	The optimized curcumin formulations should be explored as an alternative to topical antibiotics in the treatment of wound infections, as it has very high phototoxicity towards both *E. faecalis* and *E. coli*, making these lyophilizes suitable for in vivo PDT.	[63]
10	Antimicrobial action of photodynamic therapy on *Enterococcus faecalis* biofilm using curing light, curcumin, and riboflavin	Blue LED is associated with 10 µL of curcumin at 460 nm in the irradiance of 60 J cm^−2^ on *E. faecalis* biofilm.	Colony-forming units, scanning electron microscopy.	Significantly reducing *E. faecalis* colony count, aPDT with curcumin and riboflavin can serve as an adjunct to routine root canal disinfection methods.	[64]
11	Effects of curcumin-mediated antimicrobial photodynamic therapy associated with different chelators against *Enterococcus faecalis* biofilms	Curcumin with different concentrations of 17 to 18% of EDTA, or HEBP irradiated with LED light in the irradiance of 75 J/cm^2^.	Colony-forming units, confocal scanning laser microscopy.	The combination of curcumin with EDTA and HEBP similarly improved the effect of aPDT on *E. faecalis* biofilms.	[65]
12	Curcumin-induced photodynamic therapy mediated the suppression of quorum-sensing pathways of *Pseudomonas aeruginosa*: An approach to inhibit biofilm in vitro	A total of 6.75 mM of curcumin at 405 nm is used in the irradiance of 10 J/cm^2^, which reduces *P. aeruginosa* biofilm more efficiently than without light, and extracellular polymeric substance production is also reduced by approximately 94% with 10 J/cm^2^ of light dose.	Crystal violet, XTT, Congo red binding assay, and confocal scanning laser microscopy.	Curcumin-mediated aPDT inhibits biofilm formation of *P. aeruginosa* through quorum-sensing pathways via the action of singlet oxygen generation, which in turn reduces the extracellular polymeric substances of the biofilm.	[66]
13	Enhancement of photodynamic bactericidal activity of curcumin against *Pseudomonas aeruginosa* using polymyxin B	*P. aeruginosa* is treated with curcumin in the presence of 0.1 to 0.5 mg/L polymyxin B and irradiated with blue LED light in the irradiance of 10 J/cm^2^.	Colony-forming units, scanning electron microscopy.	Introducing polymyxin B has the potential to enhance the effects of aPDT treatment against Gram-negative skin infections, in particular *P. aeruginosa*.	[67]
14	Evaluation of curcumin incubation time in *Staphylococcus aureus* and *Pseudomonas aeruginosa* photodynamic inactivation	Different concentrations of curcumin and *P. aeruginosa* are cultured for 24 h at 37 °C, then irradiated with blue LED light at 460 nm in the irradiance of 30 J/cm^2^.	Colony-forming units, scanning electron microscopy.	This treatment is reduced to approximately seven logs of 1 µM S. aureus after 30 min of incubation, whereas only two logs are observed for *P. aeruginosa* at a 50 µM maximum concentration.	[68]
15	Combination of curcumin photosensitizer with laser diode to reduce antibiotic-resistant bacterial biofilms	Addition of turmeric extract to the MRSA biofilm in 403 nm diode laser irradiation at 30 s, 60 s, 90 s, 120 s, and 150 s with an energy density of 13.56 J/cm^2^.	Biofilm development assay.	The addition of PS extracts for turmeric increases the effectiveness of photoinactivation of biofilm bacteria that are resistant to antibiotics.	[69]

**Table 2:** The photodynamic action of methylene blue against bacteria.

	Study	Usage of Light and Energy (J)	Experiment Parameters	Conclusion	References
1	Photodynamic therapy using methylene blue, combined or not with gentamicin, against *Staphylococcus aureus* and *Pseudomonas aeruginosa*	Different concentrations of methylene blue (0.03–7000 μg/mL), with or without GN (1–20 μg/mL), are added to planktonic cultures or biofilms, and the samples are irradiated with a Light Emitting Diode lamp (625 nm, 7 mW/cm^2^, 18 J/cm^2^).	Biofilm development assay.	This combination cannot significantly alter the photo-inactivating effect of methylene blue against *Staphylococcus aureus* biofilms but exerts a positive bactericidal effect against *Pseudomonas aeruginosa* biofilms.	[70]
2	In vivo killing of *Staphylococcus aureus* using a light-activated antimicrobial agent	Irradiation of wounds with 360 J/cm^2^ of laser light (670 nm) in the presence of 100 µL/mL of methylene blue.	Histological evaluation, and wound temperature studies.	PDT is effective with a 25-fold reduction in the total number of viable epidemic methicillin-resistant *Staphylococcus aureus* in a wound.	[71]
3	Antimicrobial photoinactivation with methylene blue of *Staphylococcus aureus*	Methylene blue is activated with red light (660 nm) of a low energy density.	Colony-forming units.	A reduction in CFU/mL of *Staphylococcus aureus* to 60% was achieved.	[72]
4	Effect of photodynamic therapy on clinical isolates of *Staphylococcus* spp.	The Staphylococcus strain (10^6^ cells/mL) is employed via PDT using 3 mM of methylene blue with a low-power 9.65 J/cm^2^ laser.	Colony-forming units.	PDT is effective in reducing the number of viable cells, such as *Staphylococcus aureus*, ranging from 4.89 to 6.83 CFU (log_10_)/mL.	[73]
5	In vitro antimicrobial photoinactivation with methylene blue in different microorganisms	A solution of 50 μM methylene blue with a wavelength of 660 nm, 100 mW, at an irradiation time of 3 min in an energy dose of 9 J for each sample.	Colony-forming units.	PDT is effective in reducing the number of viable cells, including 74.90% of *Candida albicans*, 72.41% of *Pseudomonas aeruginosa*, 96.44% of *Enterococcus faecalis,* and 95.42% of *Staphylococcus aureus*.	[74]
6	Effectiveness of antimicrobial photodynamic therapy using a 660 nm laser and methylene blue dye for inactivating *Staphylococcus aureus* biofilms in compact and cancellous bones: An in vitro study	A concentration of 0.1 mg/mL methylene blue with a wavelength of 660 nm, 40 mW, and energy doses of 3.6, 7.2, and 12 J for 90, 180, or 300 s.	Colony-forming units, and cytotoxicity assay.	Antimicrobial photodynamic therapy (aPDT) with methylene blue dye is effective in inactivating *Staphylococcus aureus* biofilms formed in compact and cancellous bone, which showed a significant reduction (log_10_ CFU/mL).	[75]
7.	Photodynamic antimicrobial chemotherapy coupled with the use of the photosensitizers methylene blue and temoporfin as a potential novel treatment for *Staphyloccocus aureus* in burn infections	A concentration of 1 mg/mL methylene blue and 50 μM and 12.5 μM of temoporfin for *Staphylococcus aureus* with a wavelength of 640 nm for 20 min.	Colony-forming units.	Antimicrobial photodynamic therapy (aPDT) with methylene blue dye resulted in a loss of *Staphylococcus aureus* viability, with a two-log reduction in bacterial viability for both light and dark conditions after 20 min exposure time. Temoporfin has a greater antimicrobial efficacy against *Staphylococcus aureus* and *Pseudomonas aeruginosa*.	[76]
8	Methylene blue internalization and photodynamic action against clinical and ATCC *Pseudomonas aeruginosa* and *Staphylococcus aureus* strains	Methylene blue in concentrations of 100, 300, and 500 μg/mL with test strains is irradiated with an LED lamp (±660 nm) at a fluence of 10 and 25 J/cm^2^.	Colony-forming units, confocal microscopy, and DNA staining.	PDT with methylene blue can decrease the growth of the tested strains in vitro, which is significantly reduced (*p* ≤ 0.01) at an average of 5.0 logs.	[77]
9	Evaluation of photodynamic therapy using a diode laser and different photosensitizers against *Enterococcus faecalis*	Solutions containing *E. faecalis* are mixed with methylene blue and malachite green and are irradiated with a diode laser (660 nm) at a distance of 1 mm with 40 mW for 30, 60, or 120 s.	Colony-forming units.	PDT treatments using methylene blue and malachite green have an antibacterial effect against *E. faecalis*, showing potential to be used as an adjunctive antimicrobial procedure in endodontic therapy.	[78]
10	Comparing the efficacy of toluidine blue, methylene blue and curcumin in photodynamic therapy against *Enterococcus faecalis*	Methylene blue is injected into the root canals and remained there for 120 s before irradiation, which was carried out for 60 s with an LED lamp emitting light in the red spectrum with a power peak at 630 nm and output intensity of 2000–4000 mW/cm^2^.	Colony-forming units, and scanning electron microscopy.	The adjunction of methylene blue-mediated PDT via a light-emitting diode to NaOCl irrigation has antibacterial efficacy against *E. faecalis*.	[79]

**Table 3:** The photodynamic action of curcumin against SARS-CoV-2.

	Study	Usage of Light and Energy Usage (J)	Experiment Parameters	Conclusion	References
1	Optimization of anti-SARS-CoV-2 treatments based on curcumin, used alone or employed as a photosensitizer	A total of 10 μM of curcumin is irradiated with laser light at 445 nm with 0.25 W/cm^2^, fluence 15 J/cm^2^, in a continuous wave and then transferred to A549 epithelial cells.	Colony-forming units, crystal violet staining.	aPDT presents a higher flexibility and a broad spectrum of activity against different viruses, which can be exploited for different purposes, ranging from blood product decontamination, and surface and liquid disinfection for applications to treat human infections.	[86]
2	Robust antimicrobial photodynamic therapy with curcumin-poly (lactic-co-glycolic acid) nanoparticles against COVID-19: A preliminary in vitro study with Vero cell lines as a model	Ten wt.% Cur@PLGA-NPs and a blue laser at an energy density of 522.8 J/cm^2^ to inactivate SARS-CoV-2 in plasma.	Scanning electron microscopy, transmission electron microscopy, Fourier transform infrared, coagulometer, Bradford method, and titration.	aPDT exhibits in vitro anti-COVID-19 activities in the treated plasma containing SARS-CoV-2 without Vero cell apoptosis and any adverse effects on plasma quality in aPDT-exposed plasma.	[87]
3	Spray-dried curcumin-loaded nanoparticles for antimicrobial photodynamic therapy	LED device for 10 min at 457 nm with a radiant exposure of 13.2 J/cm^2^.	Colony-forming units, transmission electron microscopy, confocal scanning laser microscopy, dispersibility, aerodynamic assays, and antibacterial photoactivity assay.	A curcumin-loaded nanoformulation was successfully sprayed in the lower respiratory tract, which is used to oppose severe bacterial infections, reduce antimicrobial resistance, and facilitate the treatment of chronic lung disease associated with a high risk of infection.	[88]
4	Could curcumin hydrogel for photodynamic therapy fight against SARS-CoV-2?	Curcumin hydrogel exposure to blue light with a mean wavelength of 450 nm using a microcatheter into the lung to combat SARS-CoV-2.	Colony-forming units, scanning electron microscopy.	Curcumin incorporated with the hydrogel is a suitable candidate used for the photodynamic treatment of SARS-CoV-2, cancer, wound healing, and bacterial infection.	[89]

**Table 4:** The photodynamic action of methylene blue against SARS-CoV-2.

	Study	Usage of Light and Energy (J)	Experiment Parameters	Conclusion	References
1	A novel approach of combining methylene blue photodynamic inactivation, photobiomodulation, and orally ingested methylene blue in COVID-19 management: A pilot clinical study with 12-month follow-up	The mucosal surfaces were irradiated with methylene blue at 660 nm LED light in a continuous emission mode at an energy density of 49 J/cm^2^ for photodynamic therapy.	Cycle threshold tests, and polymerase chain reaction (PCR) tests.	Methylene blue has a broad spectrum of activity, which addresses the prevailing and future COVID-19 variants and other infections transmitted via the upper respiratory tract.	[44]
2	Photodynamic disinfection of SARS-CoV-2 clinical samples using a methylene blue formulation	Concentrations of 15 μM of methylene blue and 4 to 8 min of illumination (~860 mW) at 670 nm to inactivate SARS-CoV-2.	Real-time reverse-transcription quantitative polymerase chain reaction (RT-qPCR).	Pre-treatment of lentiviral vectors with MB-PDT prevented infection to reduce viral loads in the nasal cavity and oropharynx in the early stages of COVID-19, which may be employed to curb the transmission and severity of the disease.	[90]
3	Evaluation of methylene blue-based photodynamic inactivation (PDI) against intracellular B-CoV and SARS-CoV-2 viruses under different light sources in vitro as a basis for new local treatment strategies in the early phase of a COVID-19 infection	Both 590 nm white broadband LED light and 810 nm coherent laser light at 300 mW/cm^2^ in conjugation with a low concentration of methylene blue (31 µm and 31, 1 µm) were used to inactivate intracellular B-CoV and SARS-CoV-2.	Cytotoxicity assay, viral titration, and plaque assay.	A minimum concentration of 0.0001% MB and a minimum radiation intensity of 20,000 lx leads to a 99.99% reduction in intracellular and extracellular viruses after one minute of exposure.	[91]
